# Silibinin enhanced the efficacy of albendazole in treating the muscular phase of experimental trichinellosis

**DOI:** 10.1007/s11250-025-04429-0

**Published:** 2025-05-07

**Authors:** Mennat-Elrahman A. Fahmy, Marwa Esmat, Manal Badawi, Iman R. Abdel Shafi

**Affiliations:** 1 Medical Parasitology Department, Theodor Bilharz Research Institute (TBRI), Giza, Egypt; 2 Department of Medical Parasitology, Faculty of Medicine, Misr University for Science and Technology, 6th October City, Giza, Egypt; 3 Department of Pathology, National Research Centre, Giza, Egypt; 4 Department of Medical Parasitology, Faculty of Medicine, Cairo University, Cairo, Egypt

**Keywords:** Trichinellosis, Mice, Albendazole, Silymarin, Combination, PD1

## Abstract

*Trichinella spiralis* is a parasitic nematode with a special life cycle. Both adults and larvae live in two different niches in the same host (intestinal and muscular). The parasite is known to manipulate the immune system of the host to be able to survive. One of the pathways the parasite modulates is the programmed death 1/ programmed death ligand 1 (PD1/PDL1), a pathway important to maintain the immune homeostasis during chronic infections and cancers. Albendazole (ABZ) shows anti-trichinellosis efficacy, especially against the intestinal phase of the infection. In an attempt to discover a drug that would enhance the efficacy of ABZ against the muscular phase, we used 40 CD1 Swiss-Albino male mice divided into 5 groups: normal, infected, infected ABZ-treated, infected Silymarin (SM)-treated, and the infected-treated with a combination group. After euthanasia, the number of diaphragmatic larvae was estimated in the infected and the infected-treated groups. In addition, the tongues and hearts of all mice were subjected to histopathological and immunohistochemical processing and evaluation. Monotherapy groups showed a significant reduction of both larval count and PD1 local expression compared to the infected-only group, however, neither ABZ nor SM alone could reduce the inflammation accompanying infection. The most significant improvements were recorded in the combined treatment group with a reduction rate of 69.95%, a significant reduction of inflammatory infiltrates (*p < *0.05), and significant modulation of PDL1 local expression (*p < *0.05). So, Silibinin (the major active ingredient of SM) showed anti-trichinellosis activity and enhanced the efficacy of ABZ against the muscular phase of the infection.

## Introduction

Trichinellosis is a parasitic helminthic infection acquired by humans through ingesting improperly cooked meat infected with the infective larvae. The most common species infecting man is *Trichinella spiralis.* After ingesting the infected meat, the 1 st stage larvae are released in the stomach, invade the small intestine, and develop into adults. The female then lays newborn larvae (NBL) which enter the lymphatic circulation, and then into the blood to reach skeletal muscles, finally forming a nurse cell (NC). *Trichinella* possesses a complicated, unique life cycle with both the adults and larvae surviving in two different intracellular niches in the same host; each of these life phases affects the host immune system. To establish a chronic infection and communication with the host, the parasite induces immunomodulation through *Trichinella*-derived molecules (the adult excretory-secretory products (AES) and the muscle larvae excretory-secretory products (MES) (Sun et al. 2019; Bruschi et al. 2022).

One of the immune pathways modulated by the excretory-secretory molecules is the PD1/PDL1 pathway. Programmed cell death 1 (PD- 1) and its most important ligand, Programmed death-ligand 1 (PD-L1), which is expressed by T cells, B cells, dendritic cells, macrophages, and tumoral cells, are considered a critical immune checkpoint that modulates the T-cell function and promotes macrophage polarization. Helminths such as *Trichinella* and protozoa such as Leishmania are known to induce the PD1 pathway to modulate the host immune response and ensure their survival. The binding of PD1 to PDL1 and the formation of the PD- 1/PD-L1 complex is a mechanism used by pathogens to evade the immune response or to regulate tissue damage mediated by the immune system (Wang et al. 2020; da Fonseca-Martins et al. 2021). Blocking the PD- 1/PD-L1 signaling pathway resulted in partial restoration of both CD4 + and CD8 + T-cell exhaustion and reduction of splenic parasite load during visceral leishmaniasis (He et al. 2024). So, targeting the PD1/PDL1 axis would become a new target for treating chronic infections.

Silymarin (SM) is extracted from the *Silybum marianum* (milk thistle) plant, containing various flavonolignans (the major one is silybin) and described for liver diseases with antioxidant, anti-inflammatory, and anti-fibrotic activities in addition to a good safety profile. It showed antiparasitic properties against *Schistosoma mansoni* and *Leishmania major*. One of the targets of SM is PDL1 expression (Surai 2015; Faridnia et al. 2018).

Our work aimed at testing the possible in vivo efficacy of SM against the muscle larvae of *Trichinella spiralis* and studying the effect of the infection and the treatment (monotherapy or combined therapy) on the PD/PDL1 axis.

## Materials and methods

### Ethical approval

All the experimental animal procedures performed in the study were approved by the Ethical Committee of Theodor Bilharz Research Institute according to the National Institutes of Health (NIH) guide for the care and use of laboratory animals (eighth edition) (Approval number: PT 813).

### Preparation of the infection and adjustment of the dose

*Trichinella spiralis* strain was maintained in the muscles of the infected mice in TBRI, Giza, Egypt. The previously infected mice were euthanized by intraperitoneal injection of an anesthetic-anticoagulant solution (500 mg/kg thiopental and 100 units/mL heparin) (Laferriere and Pang 2020) and skinned. The muscles of each mouse were isolated and cut into tiny pieces, then exposed to artificial digestion using a pepsin-HCl mixture, thieved, washed, and the larvae were recovered and collected to be examined under the microscope (× 40). The infection dose was then adjusted to about 200 larvae per mouse (Gajadhar et al. 2019).

### Experimental animals and study design

A total of 40 CD1 Swiss-Albino male mice (aged 6–8 weeks, and weighed 20 - 25 gm each) were purchased from TBRI and divided into 5 groups (8 mice each); G1: The non-infected group, G2: The infected non-treated group, G3: The infected group treated with Albendazole (ABZ), G4: The infected group treated with SM, and G5: The infected group treated with a combination of ABZ and SM. The animals were housed in plastic cages, each group in a cage with free access to food and water.

### The infection

Each mouse (in the infected and the infected-treated groups) was infected with an average of 200 *T. spiralis* larvae in 200 µl of phosphate buffer saline (PBS) through oral gavage. While mice in the normal control group received the same amount of oral PBS.

### The treatment

The treatment regimens for the infected-treated groups started orally after 35 days post-infection via an oral gavage tube. For each mouse in G3 and G5, the reference drug (ABZ) in the syrup form (Bendax, SIGMA Pharmaceutical Industries, Egypt) was received at a dose of 250 mg/kg for 6 consecutive days (Li et al. 2012). Meanwhile, each mouse in G4 and G5 received SM in gelatinous capsules (Silymarin plus, South Egypt Drug Industries Co., SEDICO) at a dose of 750 mg/kg for 5 consecutive days (El-Lakkany et al. 2012, with modification).

### Mice euthanasia and tissue preparation

All mice were euthanized using an anesthetic-anticoagulant solution as described above after the last day of treatment. Diaphragms of the infected and the infected-treated groups were excised carefully and subjected directly to examination and larval counting under the microscope (× 40) after pressing between two glass slides (Basyoni and El-Sabaa 2013; Li et al. 2019), and the reduction rates were calculated according to Gu et al. (2020). Each mouse's cardiac tissues and tongue in the study groups were preserved in 10% formalin for either histopathological processing or immunohistochemical staining.

### Histopathology and immunohistochemistry processing and evaluation

Specimens in formalin 10% were then processed and embedded in paraffin. Some sections from each tissue were then exposed to staining with hematoxylin and eosin to be evaluated under the microscope (Slaoui and Fiette 2011). Histopathological evaluation of inflammatory cellular infiltration was classified according to Li et al. (2020) into no, minimal, mild, moderate, and severe based on the percentage of inflammatory cells compared to the control group within 5 fields of vision (× 100). Other sections obtained from the formalin-fixed paraffin-embedded specimens were subjected to a series of steps, including staining with Mouse Monoclonal Anti-PD- 1 Antibody (Sino Biological, Inc., Beijing, China). Quantitative analysis of the area percentage of the PD1-positive cells within 5 fields of vision (× 100) was performed at the Pathology Department, National Research Centre, Giza, Egypt, using Leica Qwin500 Image Analyzer (Cambridge, England).

### Statistical analysis of data

The number of diaphragmatic larvae and the local muscular expression of PD1 in each study group were represented by quantitative data (Mean ± SD), while the inflammatory score was recorded as qualitative data. All analyses were performed using SPSS software version 28.0 (IBM Corp, Armonk, NY). One-way analysis of variance (ANOVA) tests, followed by LSD post hoc test in addition to chi-square tests, were performed for multiple pairwise comparisons between the study groups. A value of *P < *0.05 was considered statistically significant.

## Results

### The number of diaphragmatic larvae decreased significantly in all treated groups

All treatment regimens used in this study reduced the larval burden significantly compared to the infected non-treated group. The combined therapy group reported the lowest number of larvae (75.625 ± 14.5) with the best reduction rate (69.95%). Meanwhile, monotherapy groups showed lower reduction rates (63.68% and 46%) for ABZ-treated and SM-treated groups, respectively (Table 1). However, there was no significant difference between the reduction rates recorded in groups 3 and 5.

**Table 1:** Showed the mean diaphragmatic larval count in each group and the reduction rates among groups. One-way ANOVA test and LSD post hoc test were used for pairwise comparisons a, b, and c: groups with the same subscript letter have no significant difference

Groups	Mean ± SD	Reduction rates	*P* value
G2	251.66 ± 24.3^a^		*P < *0.001**
G3	91.4 ± 19.5^c^	63.68%	
G4	135.7 ± 44.29^b^	46%	
G5	75.625 ± 14.5^c^	69.95%	

### The combination therapy significantly improved the histopathological changes caused by the infection, reduced the inflammation, and downregulated PD1 expression in the tongue muscles

**Fig. 1:**
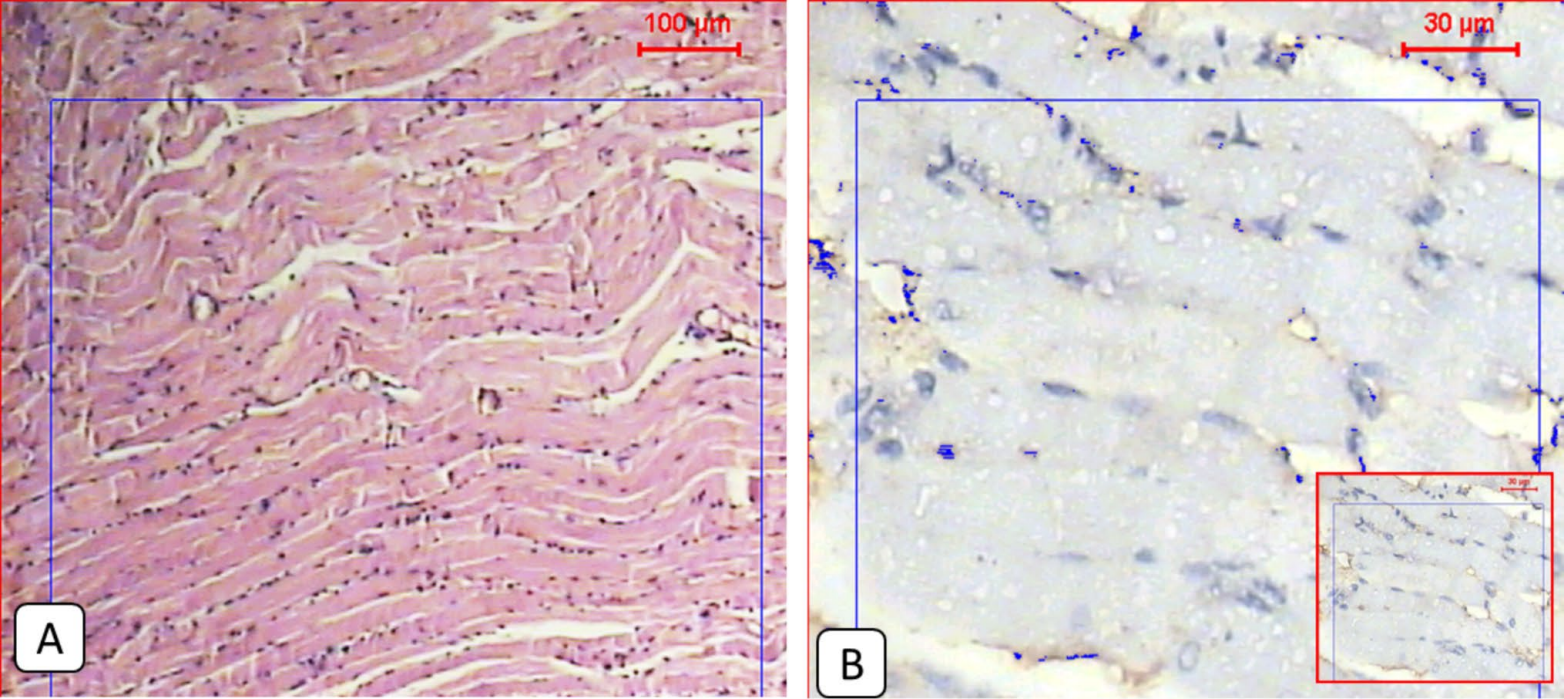
**A**: Light microscopic picture of the tongue muscle section of non-infected mice showed normal longitudinal muscle fibers (H&E 100x). **B**: Light microscopic picture of the immunohistochemical staining during the real-time digital analysis of a normal muscle section showed very low expression of the protein marker

**Fig. 2:**
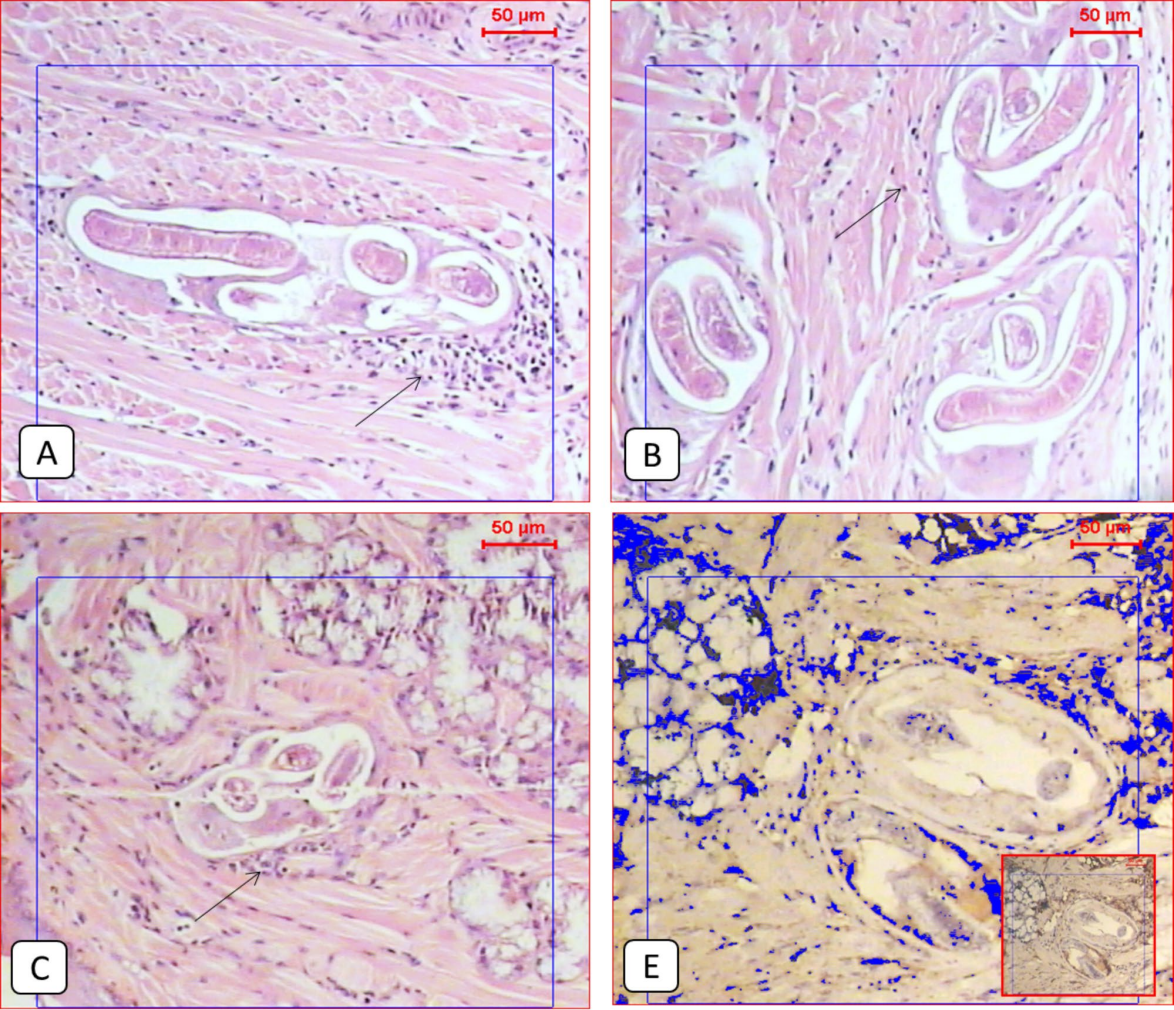
**A** & **B**; Histological examination of the tongue tissue showed features of a differential inflammatory response around *Trichinella* larvae in the infected non-treated group (black arrows) (H&E, X100). **C**: *Trichinella* larvae near the salivary glands of the tongue (H&E, X100). **E**: Light microscopic picture of the immunohistochemical staining of the infected non-treated group showed a relatively high expression of the protein marker (IHC, X100)

**Fig. 3:**
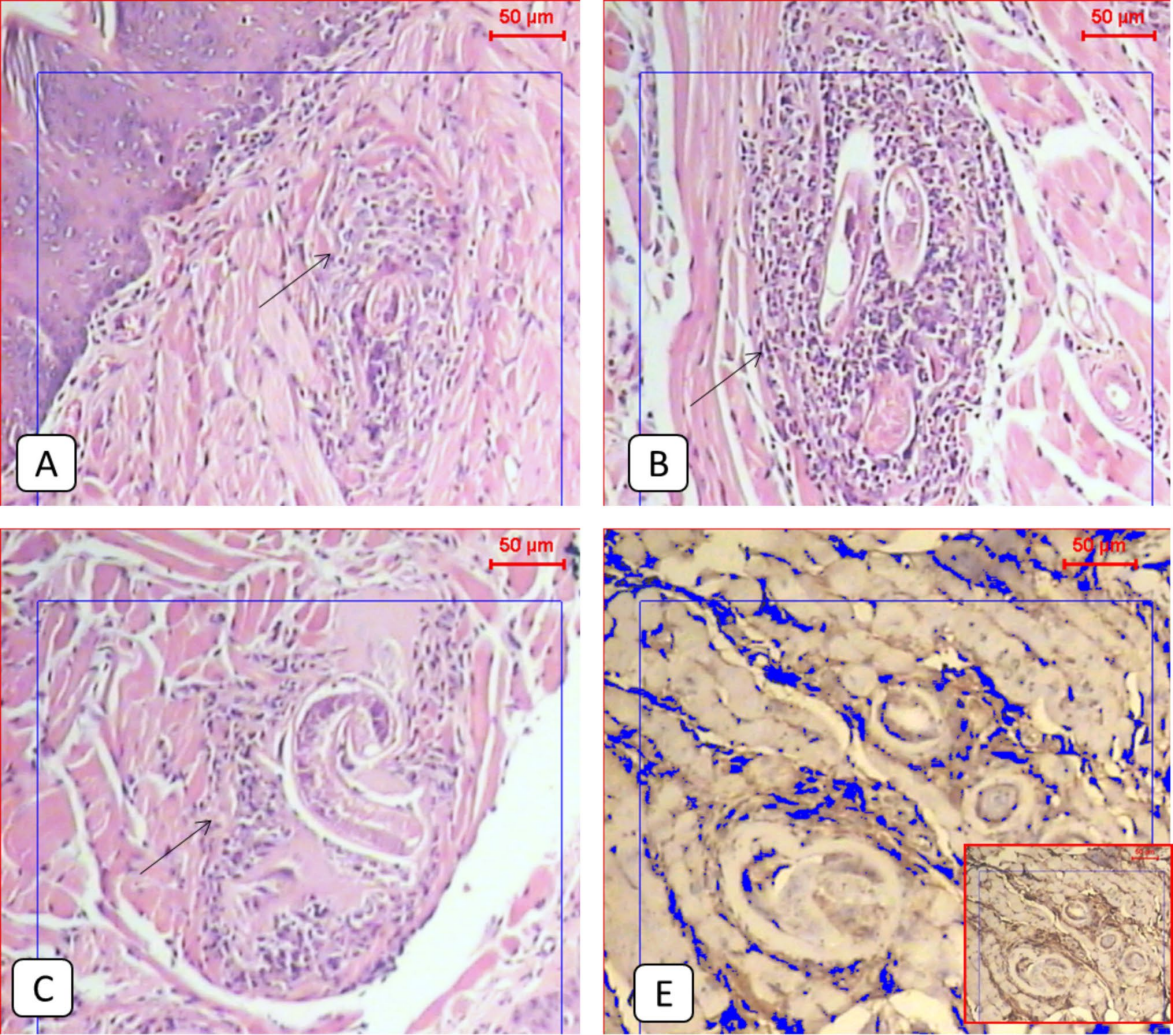
**A**, **B** & **C**: Histological examination of the tongue tissue showed extensive inflammatory response around almost destructed *Trichinella* larvae in the infected treated group with ABZ (black arrows) (H&E, X100). **E**: A light microscopic picture of the immunohistochemical staining of the infected non-treated group showed moderate expression of the protein marker (IHC, X100)

**Fig. 4:**
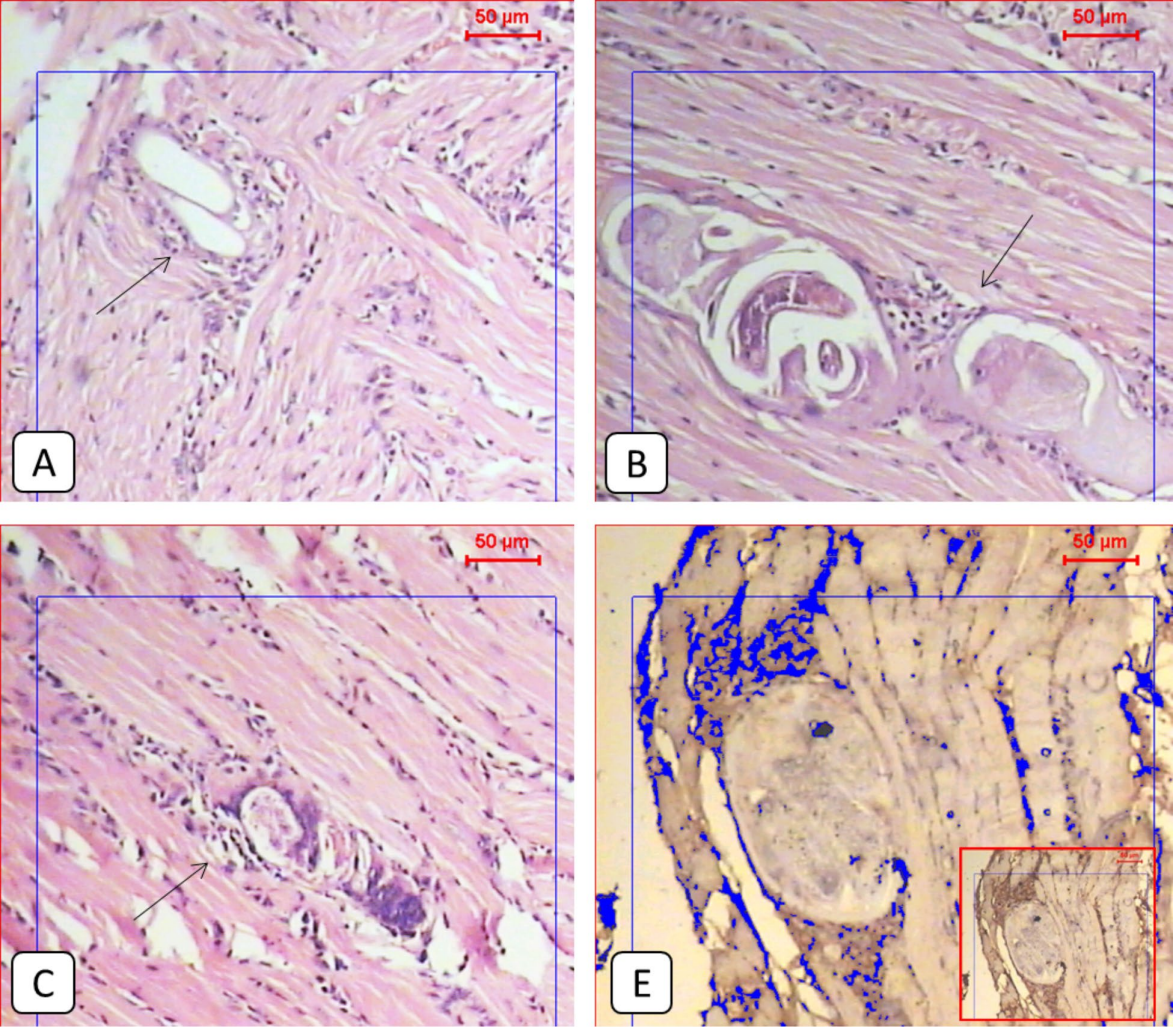
**A**, **B** & **C**: Histological examination of the tongue tissue showed a mild inflammatory response around almost destructed *Trichinella* larvae in the infected treated group with SM (black arrows) (H&E, X100). **E**: Light microscopic picture of the immunohistochemical staining of the infected non-treated group showed adequate expression of the protein marker (IHC, X100)

**Fig. 5:**
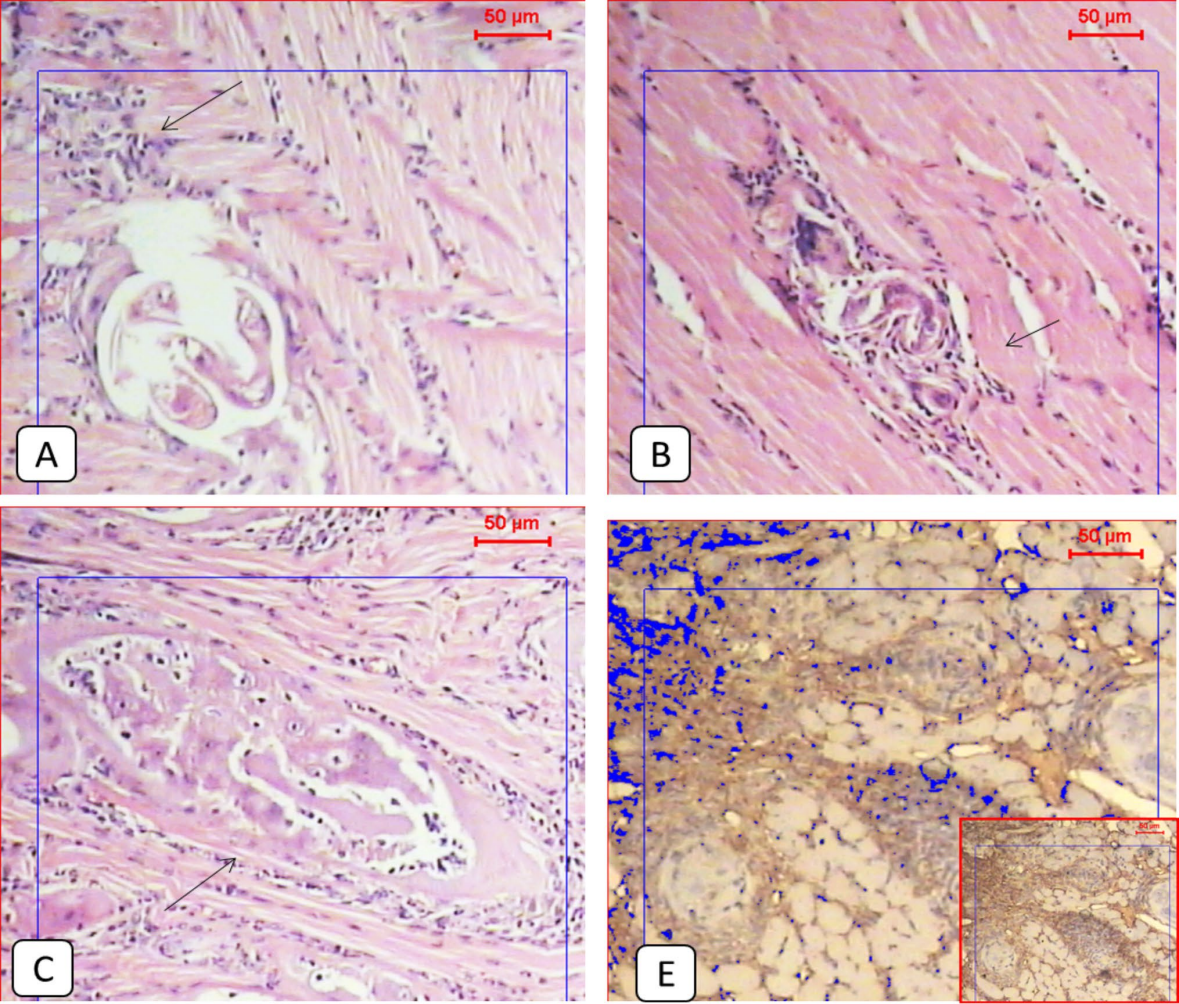
**A**, **B** & **C**: Histological examination of the tongue tissue showed a mild inflammatory response around almost destructed *Trichinella* larvae in the infected treated group with combined ABZ plus SM (black arrows) (H&E, X100). **E**: A Light microscopic picture of the immunohistochemical staining of the infected non-treated group shows moderate expression of the protein marker (IHC, X100)

**Fig. 6:**
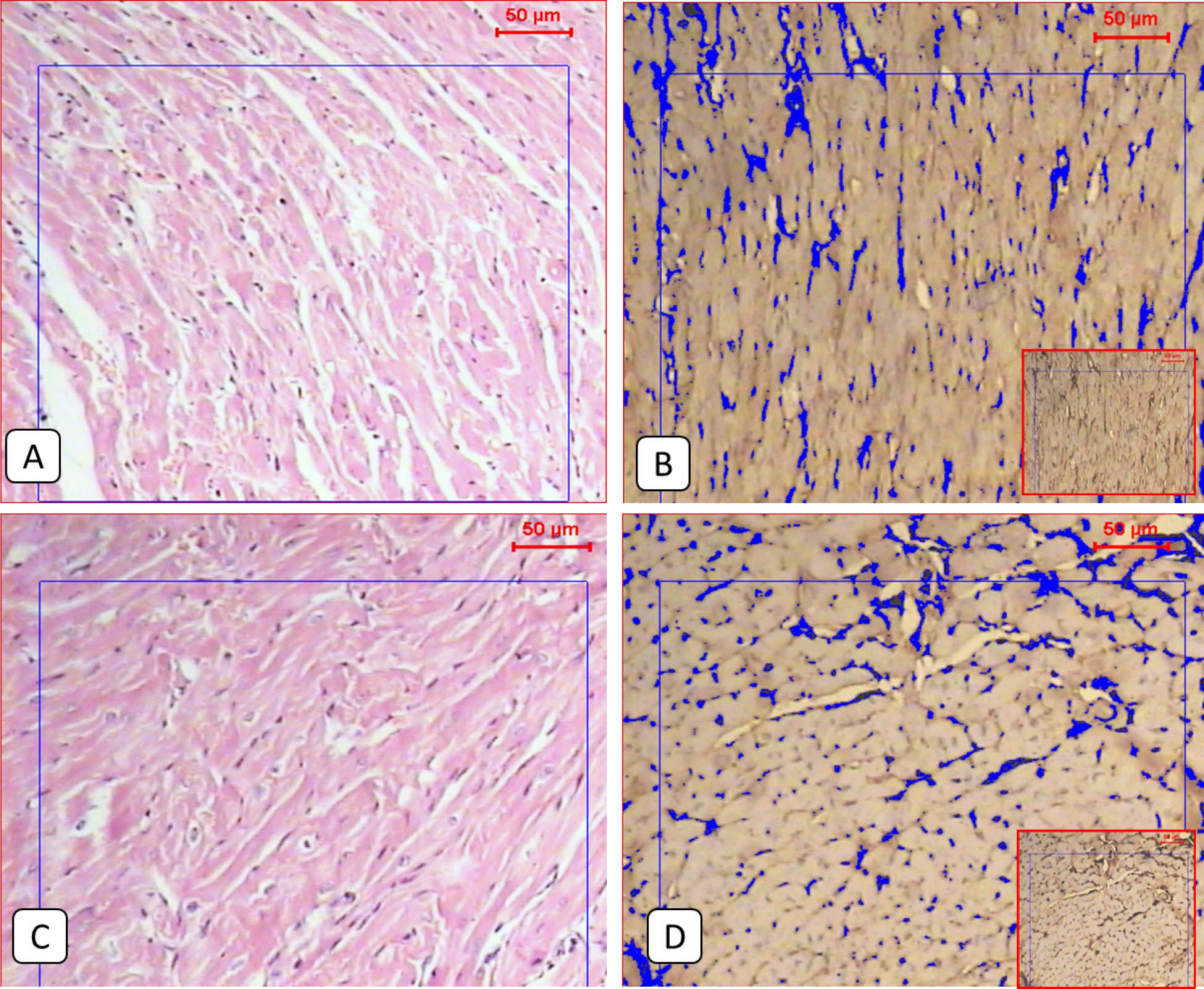
**A** Representative light microscopic pictures of the cardiac muscle section of the infected non-treated group showed normal muscle fibers (H&E 100x). **B**: Light microscopic picture of the immunohistochemical staining of the infected non-treated group showed moderate expression of protein marker (IHC 100x). **C**: The Cardiac muscle section of the infected-treated group showed normal muscle fibers (H&E 100x). **D**: related IHC staining picture showed moderate expression of protein marker (IHC 100x). IHC cardiac expression is shown to be nearly the same as expressed within the skeletal muscle fibers, regardless of being clear of parasitic stages

Compared to the normal appearance of the tongue muscles and the very low PD1 expression seen in the non-infected group (Figs. 1 and 2), the infected non-treated group showed severe infection with encysted larvae surrounded by thick intact capsules and moderate to severe inflammatory infiltration accompanied by high local PD1 expression (Figs. 1 and 3 and Table 2). The ABZ-treated group showed fewer encysted larvae with moderate inflammation, and some of the larvae were partially destructed with severe inflammatory infiltrates, insignificantly different from the infected group (Fig. 4 and Table 2). A moderate PD1 local expression was also observed (Figs. 1 and 4). However, the SM-treated group showed significantly less inflammatory infiltrations with mild to moderate marker expression (Figs. 1 and 5 and Table 2). The most significant histopathological and immunohistochemical improvements were detected in the combination therapy group in the form of a significant reduction of larvae, inflammation, and local expression of PD1 (Figs. 1 and 6 and Table 2).

**Table 2:** Showed the inflammatory score in each study group. The chi-square test was used for pairwise comparisons. Statistically significant differences (*P < *0.05) are denoted by different letters. Means with the same letter indicate no statistically significant differences (*P >* 0.05)

Groups		Inflammatory infiltrates					Total	χ^2^	*P*-Value
		None	Minimal	Mild	Moderate	Severe			
G1^c^	N	7	1	0	0	0	8	51.264	< 0.001**
	%	87.5%	12.5%	0.0%	0.0%	0.0%	100.0%		
G2^a^	N	0	0	0	2	4	6		
	%	0.0%	0.0%	0.0%	33.3%	66.7%	100.0%		
G3^a^	N	0	0	0	4	3	7		
	%	0.0%	0.0%	0.0%	57.1%	42.9%	100.0%		
G4^a, b^	N	0	0	2	4	1	7		
	%	0.0%	0.0%	28.6%	57.1%	14.3%	100.0%		
G5^b^	N	0	2	3	3	0	8		
	%	0.0%	25.0%	37.5%	37.5%	0.0%	100.0%

### The cardiac muscles showed only immunohistochemical changes in all study groups

**Fig. 7:**
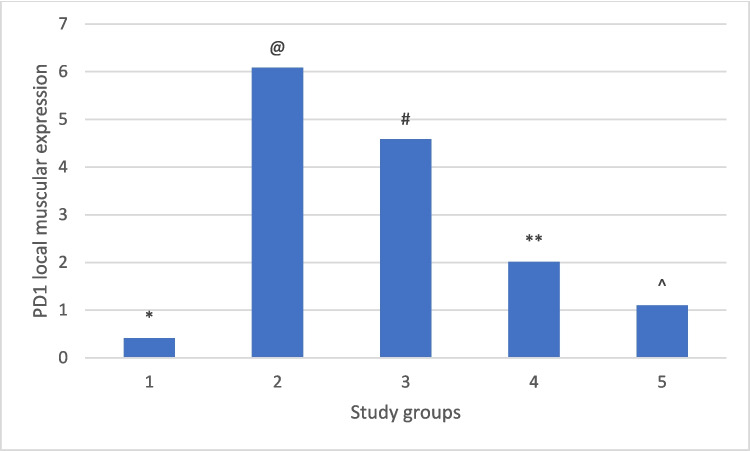
Showed the mean PD1 local muscular expression among different study groups. One-way ANOVA test and LSD post hoc test were used for pairwise comparisons. The groups showing the same symbol have no significantly different values

Although no histopathological changes in the cardiac muscles were detected in all study groups, the same alterations observed in the local expression of PD1 in the tongue muscles were detected in the cardiac muscles (Fig. 7).

## Discussion

In the absence of an effective drug against the muscular phase of trichinellosis, Evaluating the possible anti-*Trichinella spiralis* efficacy of silymarin (the active ingredient of the milk thistle plant known to have a modulatory action on PDL1 protein) alone or combined with Albendazole (the reference drug) for the first time was the aim of our study. The study relied on several parameters: the diaphragmatic larval count, the muscular histopathological changes, and the alterations in the cardiac and skeletal muscle expression of the PD1 protein.

Regarding the larval count observed in the diaphragm of each mouse in the infected and infected-treated groups, significant reduction rates were reported in all treated groups: ABZ-treated, SM-treated, and combination-treated, compared to the infected non-treated group (63.68%, 46%, and 69.95%, respectively). Similarly, Li et al. (2012) reported a reduction rate of 62.62% in the diaphragms of mice infected with *Trichinella spiralis* after administration of the same dose of ABZ. However, Siriyasatien et al. 2003 reported a reduction rate of 71% after using ABZ after 30 days of infection at 20 mg/kg for 30 days, while Chai et al. 2021 reported other studies with effective reduction rates after using 50 mg/kg for 5 days and trying different combinations with ABZ. Observing different results could be explained by using different treatment dosages and regimens.

In accordance with our results, El-Lakkany et al. (2012) also tried SM alone against *Schistosoma mansoni* infection in mice and found remarkable worm and egg reduction rates associated with the healing of hepatic granulomas. Similar results of significant reduction rates of parasitic burden after using a combination of SM with other anti-helminthics were obtained by Hrckova and Velebny (2013) who documented a significant reduction rate of encysted hepatic larvae of *Mesocestoides vogue *in vivo after receiving combination therapy of praziquantel and SM, and El-Lakkany et al. (2012) who reported a complete eradication of *Schistosoma mansoni* worms and reduction in the hepatic tissue egg loads after using the same combination of SM and praziquantel at different time intervals. Working on different parasites, different experimental animals, and differences in the dose, duration, and combination of the administered drugs should be the reasons behind the different reduction rates documented from our study.

In our study, the tongue muscles of the infected non-treated mice showed intact larvae surrounded by moderate to severe inflammatory infiltration and significantly higher local expression of PD1 compared to other study groups. The level of inflammation around the nurse cells was similarly reported by Park et al. (2018), who stated that around 6 weeks of *T. spiralis* infection was the peak of the significant increase in the inflammatory infiltrates, which gradually decreased after that. However, Esmat et al. (2021) and Eissa et al. (2022) reported histopathological moderate inflammatory infiltrates around the larvae, which may be due to the different timing of euthanasia of mice post-infection. Concerning the significantly high PD1 local muscular expression reported in our study, Similar results were obtained by Cheng et al. (2018) who reported increased expression of PD- 1 on CD4 + T cells in the spleens of mice infected with *T. spiralis*. Wang et al. 2020 also observed a significant upregulation of PD- 1 expression in macrophages of mice treated with excretory/secretory products from *Trichinella spiralis* adult worms (AES). Both studies described the PD- 1/PD-L1 pathway as a potent inhibitory pathway induced by *T. spiralis* infection for immune regulation, keeping balanced host immune responses during the infection, and minimizing inflammatory tissue damage. Sharpe and Pauken (2018) described a high sustained increase in PD1 expression levels due to persistent antigen exposure during chronic infections and cancer.

Our results revealed histopathological changes in the ABZ-treated group in the form of a decreased number of encysted larvae and moderate inflammation. Some larvae showed partial destruction with areas of extensive inflammatory infiltrates around them. In addition to moderate PD1 local muscular expression. Many authors similarly described the damage of *Trichinella* larvae by ABZ in vivo and in vitro (Chen et al. 1999; Li et al. 2012; Abd-Elrahman et al. 2020). In contrast, Ibrahim et al. (2024) reported intact nurse cells but still accompanied by moderate inflammatory infiltrates after using a different dose and duration of ABZ. The reason behind the moderate inflammation observed in our work after ABZ is that the destruction of larvae exposes them to the immune system, and the increase in the number of immune cells caused by ABZ treatment itself, as stated by Ricken et al. (2017). Zhu et al. (2022) described ABZ as an anti-PD1 therapy that decreased tumor PDL1 expression through the induction of ubiquitin-mediated PD-L1 protein degradation, making ABZ a promising antitumor therapy candidate.

SM alone in our results caused less inflammatory reaction and a decrease in the PD1 local muscular expression. These results were in agreement with the anti-inflammatory properties of SM reported by Wadhwa et al. (2022). Koltai and Fliegel. (2022) also described a significant reduction in both PD1 expression in HIV-positive CD4^+^ T cells and PD-L1 expression in cancer cells by Silymarin. The modulatory action of SM on the PD1/PDL1 pathway was described by Cuyàs et al. (2016), who revealed a reduction of the mRNA expression of PD-L1 through inhibiting STAT3 phosphorylation. In addition, Sellam et al. (2020) explained the ability of silibinin to inhibit PDL1 expression in vitro by suppressing HIF- 1α/lactate dehydrogenase (LDH-A)-mediated aerobic glycolysis.

The best histopathological improvement in our study was observed in the group that received the combination treatment associated with low PD1 local expression. Increasing the efficacy of ABZ by using medically important herbs has been reported by previous studies (El-Kady et al. 2022; Albogami 2023). In our work, the combination achieved the best results; high-dose ABZ killed the larvae directly, increased the inflammatory cells, and modulated the PD1 local expression while Silymarin showed anti-inflammatory, antioxidant, and anti-angiogenesis properties in addition to the modulatory action on PD1 expression which enhanced the efficacy of ABZ and created a balanced immune response against the infection with significant reduction of the larval burden, and regression of the resultant myositis.

Interestingly, the histopathological changes observed in our study were only found in the skeletal muscles of the tongue. In contrast, the cardiac muscles were free from histopathological changes in all study groups. However, the PD1 expression alterations were seen the same in both the tongue and cardiac muscles, even with the absence of muscle larvae. The inability of *T. spiralis* to encyst in cardiac muscles was also reported by Paolocci et al. (1998). Yan et al. (2021) described the cardiac involvement in trichinellosis as an immunopathological process, which could be the explanation for our findings. On the contrary, Albogami (2023) reported encysted larvae in the cardiac muscles. Using a mouse strain and infecting dose different from our study may be the cause.

Despite the anti-*Trichinella* effects of SM observed in our study, especially when combined with Albendazole, using PD1 modulators should be prescribed with caution. Patients receiving immune checkpoint inhibitors (ICI) may develop ICI-associated myocarditis (Mahmood et al. 2018). In addition, inhibition of PD- 1 or its ligands by anti-PD- 1 or PD-L1 inhibitors could result in muscle weakness, asthenia, and myasthenic-like syndrome (Sun et al. 2020).

Adjusting the dose and careful evaluation of the immunological status of the patient are vital for the safe and effective usage of PD1/PDL1 pathway modulators in treating trichinellosis.

## Conclusion

Silymarin, the extract from milk thistle seeds (*Silybum marianum*), has shown anti-trichinellosis efficacy. Silymarin enhanced the efficacy of Albendazole in treating trichinellosis by reducing the larval burden, alleviating inflammation, and modulating the PD1/PDL1 pathway.

## Data Availability

The data that support the findings of this study are available from the corresponding author (Mennat-Elrahman Ahmed Fahmy), upon reasonable request.
